# Risk factors for poor prognosis in adult outpatient urinary tract infection: a meta-analysis

**DOI:** 10.3399/BJGPO.2024.0298

**Published:** 2025-09-24

**Authors:** Peter K. Kurotschka, Felix Kannapin, Andreas Klug, Maria Chiara Bassi, Ildikó Gágyor, Mark H. Ebell

**Affiliations:** 1 University Hospital Wurzburg, Department of General Practice, Wurzburg, Germany; 2 Medical Library, Azienda USL-IRCCS Reggio Emilia, Reggio Emilia, Italy; 3 Department of Family Medicine, College of Human Medicine, Michigan State University, East Lansing, Michigan, United States

**Keywords:** Urinary tract infections, Prognosis, General practice, Cystitis, Pyelonephritis, Outpatients

## Abstract

**Background:**

Risk factors for poor prognosis in outpatient urinary tract infection (UTI) vary across studies and clinical guidelines.

**Aim:**

To review the evidence on risk factors for poor prognosis in adults’ UTI.

**Design & setting:**

Systematic review and meta-analysis of observational studies performed in the outpatient setting.

**Method:**

Five databases and citations of included studies were searched. Two reviewers independently screened studies, abstracted data, and assessed risk of bias (RoB). Random-effects meta-analysis of relative risks (RR) and adjusted odds ratios (aORs) were performed for risk factors reported by ≥3 studies.

**Results:**

Thirty-five cohort studies including 1 532 790 adults with cystitis or pyelonephritis (PN) were included. Ten were at moderate to high RoB. Increasing age was the only independent predictor of re-consultation (aOR 1.18 per decade). Hospitalisation was associated with high procalcitonin (PCT) (aOR 5.12), increasing age (aOR 3.51 if aged ≥65 years; aOR 1.27 per decade), hypotension (aOR 3.29), fever >38°C (aOR 2.08), elevated C-reactive protein (CRP) (aOR 1.62), creatinine ≥1.2 mg/dl (aOR 1.56), male sex (aOR 1.41), and diabetes (aOR 1.34). In the only study on mortality, among patients aged ≥65 years with cystitis, this outcome was associated with no antibiotics; older age; hospitalisation or antibiotics in prior month; higher comorbidity index; and smoking.

**Conclusion:**

Older age, male sex, elevated CRP, and diabetes are predictors of adverse outcomes in both patients with cystitis and PN. Elevated PCT, creatinine, hypotension, and fever predict hospitalisation in patients with PN only. These findings support risk stratification and patient management, but further studies are needed to consolidate knowledge on risk factors, especially for patients with cystitis.

## How this fits in

The evidence supporting risk stratification for adverse outcomes in outpatients with urinary tract infections is fragmented. This meta-analysis consolidates key predictors of poor prognosis, including advanced age, clinical signs, elevated inflammatory biomarkers, diabetes, male sex, and elevated creatinine. These findings equip clinicians with evidence-based factors to guide individualised management for higher-risk patients, optimising care and resource allocation. However, especially for risk factors of adverse outcomes in patients with acute cystitis, further longitudinal studies are needed to strengthen the evidence base.

## Introduction

Urinary tract infection (UTI) is the most common bacterial infection encountered in the outpatient setting.^1^ The spectrum of UTI ranges from a mild condition of the lower urinary tract (acute cystitis), to a more severe infection affecting the upper urinary tract (acute pyelonephritis [PN]), to severe sepsis, which has a mortality rate of up to 40%.^2^ Acute cystitis is typically a mild condition, easily treated with a short course of antibiotics and/or anti-inflammatories,^3^ while PN always requires prompt antibiotic treatment.^4^ For patients classified as being at higher risk of complications, current practice guidelines suggest further imaging, urine culture, blood tests, close follow-up, and possibly hospital admission.^4,5^

Adverse disease outcomes in patients with UTI include the need for repeated consultations, hospital admission, or bloodstream infection (sepsis).^6^ Risk factors for these adverse outcomes include immunocompromise, increasing age, diabetes mellitus (DM), recent catheterisation or instrumentation, and functional or anatomical urinary tract abnormalities.^7,8^ Other factors associated with unfavourable clinical outcomes include male sex, previous healthcare exposure, prior antibiotic use, and a history of recurrent UTIs.^9–11^ Additionally, specific symptoms (for example, urgency or frequency) and abnormal blood or urine test results have also been linked to poorer outcomes.^9–16^ However, these factors have been identified inconsistently across studies, with sometimes conflicting results.

This inconsistency is reflected in current management guidelines that differ in their definition of risk factors for a complicated course in patients with UTI.^17^ The definition of complicated UTI itself varies widely, often reflecting factors such as the risk of treatment failure, infection severity, or anatomical location.^4,17,18^ Complicated UTIs are commonly associated with indwelling catheters, systemic symptoms indicating infection beyond the lower urinary tract, or host factors such as, structural abnormalities, immunosuppression, or comorbidities.^3,4,7,18–24^ Moreover, male sex has historically been regarded as a criterion for complicated UTI, although this is controversial and lacks supporting evidence except in cases involving prostate infections.^17,25^

To inform guideline recommendations, clinical management and future research in the field, we performed a systematic review and meta-analysis of observational studies to identify the most important independent predictors of poor prognosis in adult outpatients with UTI.

## Method

This systematic review was prospectively registered in PROSPERO (CRD42022355676) and is reported according to the Preferred Reporting Items for Systematic Reviews and Meta-Analyses (PRISMA) 2020 guidelines.^26^

### Search strategy

Using multiple terms for ’urinary tract infection’ *and* (’general practice’ *or* ’emergency’ *or* ’outpatient’) *and* (’risk factor*’ *or* ’predictive value’ *or* ’sensitivity and specificity’ *or* ’prognosis’) a professional information specialist (MCB) searched MEDLINE (via PubMed), Embase, CINAHL, Scopus, and Web of Science from inception to 24 October 2022, with an update on 3 November 2023 (see Appendix A, Supplementary Table S1, for the full search strategies). Reference lists of included studies were also reviewed.

### Inclusion criteria

Studies had to initially diagnose UTI in the outpatient setting (primary care, subspecialist ambulatory care, or emergency department [ED]), and they had to report on women or men aged ≥14 years with a UTI (acute cystitis and/or PN), and on at least one risk factor for poor prognosis. Studies had to report sufficient data to either calculate univariate relative risks (RRs) or summary effect estimates such as adjusted odds ratios (aORs) with corresponding 95% confidence intervals (CIs) for multivariable models. Moreover, studies had to report on at least one adverse clinical outcome, such as the need for follow-up consultation, recurrence within 30 days, hospitalisation, bacteremia, sepsis, septic shock, admission to the intensive care unit, or mortality. We limited potential risk factors to demographics, comorbidities, vital signs, symptoms, and laboratory tests judged to be available in at least some outpatient settings.^27–30^ The latter included blood or urine cell counts, dipstick tests, C-reactive protein (CRP), procalcitonin (PCT), white blood cell count, and blood chemistry. See Appendix B (Supplementary Table S2 and S3) for details.

We included both prospective and retrospective observational studies. Studies reporting symptoms as risk factors were only included if data were collected prospectively to avoid recall and reporting biases. Randomised controlled trials (RCTs), case series, studies including children, focusing exclusively on specialised populations (for example, patients with diabetes, patients with obstructive PN, HIV-positive patients, pregnant patients, and so on), and non-peer-reviewed studies were excluded. No restrictions by length of follow-up (except for recurrence >30 days), country, or language were applied.

### Data abstraction

After de-duplication via Endnote, titles and abstracts were imported into Rayyan,^31^ and reviewed for inclusion by two blinded investigators (PK and FK). For any abstract of interest, the same two investigators reviewed the full articles in parallel against predefined inclusion and exclusion criteria, and abstracted outcome and risk factor data on Google spreadsheets. Disagreements were solved by discussion or appeal to a senior investigator (MHE, IG).

### Data preparation

Outcomes were grouped into two clinically meaningful composite categories by the lead investigators (PK, MHE), both primary care physicians, to reflect relevant clinical trajectories: (1) re-consultation, defined as the need for a follow-up visit, and (2) hospitalisation ( see Appendix C, Supplementary Table S4 for detailed definitions). Mortality was included as a secondary outcome to ensure comprehensive assessment of all patient-relevant endpoints. Similar risk factors (for example, age >65 years and ≥65 years) and cut-offs for biomarkers were grouped where it was felt to be clinically reasonable. See Appendix B (Supplementary Table S2 and S3) for details.

### Quality assessment

To assess study quality, we used the Quality in Prognostic Studies (QUIPS) tool.^32^ Low, moderate, and high-risk categories for each of the six domains were pre-specified, as well as a judgement on the overall quality of the study. Two independent investigators reviewed each included study (PK and FK or AK). Disagreements were solved by discussion or appeal to a senior reviewer (MHE).

### Data analysis

When at least three studies reported data for a risk factor, we performed random-effects meta-analysis of RRs. To summarise multivariable logistic regression models, we calculated pooled aORs from the log transformed adjusted summary effect sizes and 95% CIs.^33,34^ In cases of zero events within individual studies, we applied a continuity correction of 0.5. We created forest plots for each outcome and measured heterogeneity with the *I* ^2^ statistic and by visually inspecting the forest plots.^35^

If only two studies were available for risk factor data, we reported the range of the two effect sizes. If only one study was available, we reported the effect size with 95% CIs extracted from that study. We used the metan procedure of Stata (version 18.0)^36,37^ for analysis.

## Results

**Figure 1.**
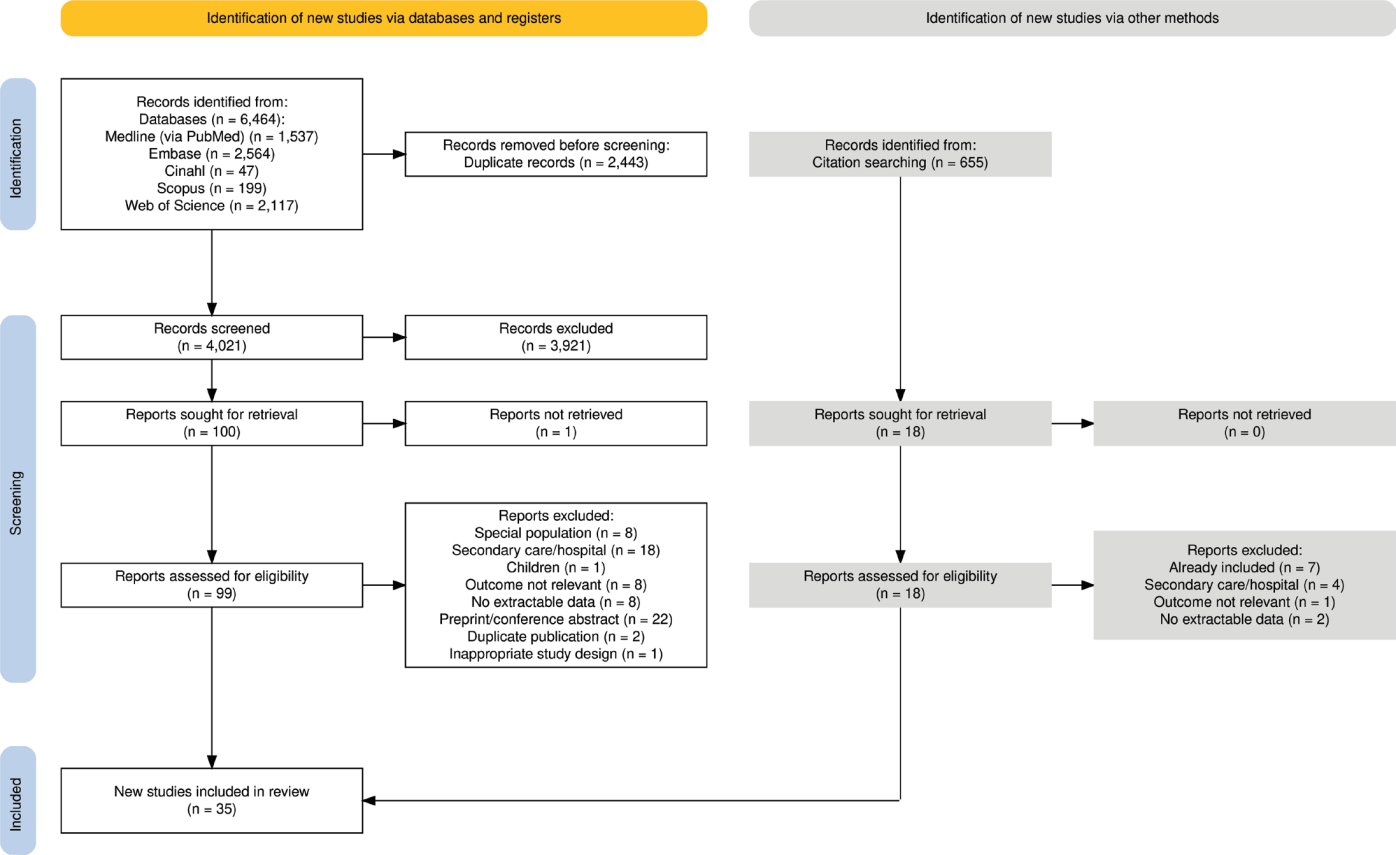
Flow diagram of included studies according to the Preferred Reporting Items for Systematic Reviews and Meta-Analyses (PRISMA 2020)^26^ guidelines. Figure drawn using the PRISMA Flow Diagram Shiny app.^73^

We identified 4676 records and reviewed 117 of them in full. We ultimately included 35 studies with 1 532 790 patients. Figure 1 shows the selection of included studies according to the PRISMA 2020 guidelines,^26^ and Supplementary Table S5 (Appendix C) lists excluded studies with reasons.

### Study characteristics

Nineteen studies included patients with PN,^10,12,15,16,38–52^ four patients with cystitis,^9,14,53,54^ and 12 included patients with either condition.^11,13,55–63^ The average age of participants ranged from 32–77 years. Twenty-five studies included women and men,^9,11–13,15,16,38–44,46,50–52,54,55,58,59,61–64^ eight only women,^10,14,44,45,47–49,60^ and two only men.^53,57^ Seventeen studies were conducted in Europe,^9,11–14,16,39,41,43,50–54,64^ four in North America,^10,40,57,58^ one in Israel,^56^ and the remainder in East Asia.^15,38,42,44–49,55,59–63^ All were cohort studies, 10 of which collected data prospectively,^13,14,16,39,41,43,45,47,50,51^ Most studies enrolled patients in the ED, while 11 recruited in general practices^9,11,16,50,53,54^ or subspecialist outpatient clinics.^10,14,15,57^ Supplementary Table S4 (Appendix C) summarises study characteristics.

### Study quality

We considered 29^9–11,14–16,38–42,44–54,56,57,60–62,64^ studies to be at moderate risk of bias (RoB) for participation because only a subset of our population of interest was included (for example, only women, or only older patients). Except for one study, which we considered at high RoB (lost to follow-up exceeded 40%),^43^ all were at low RoB for attrition, exposure ascertainment, and outcome ascertainment. Nine studies^12,13,39,43,47,50,52,55,58^ did not provide a multivariable analysis, and were classified as high RoB for confounding. Overall, we considered five studies at high overall RoB,^39,43,47,50,52^ five at moderate overall RoB,^12,13,55,58,64^ and the remainder at low overall RoB. See Appendix D in the supplementary materials for the adapted QUIPS tool and the full quality assessment.

### Outcomes

**Figure 2.**
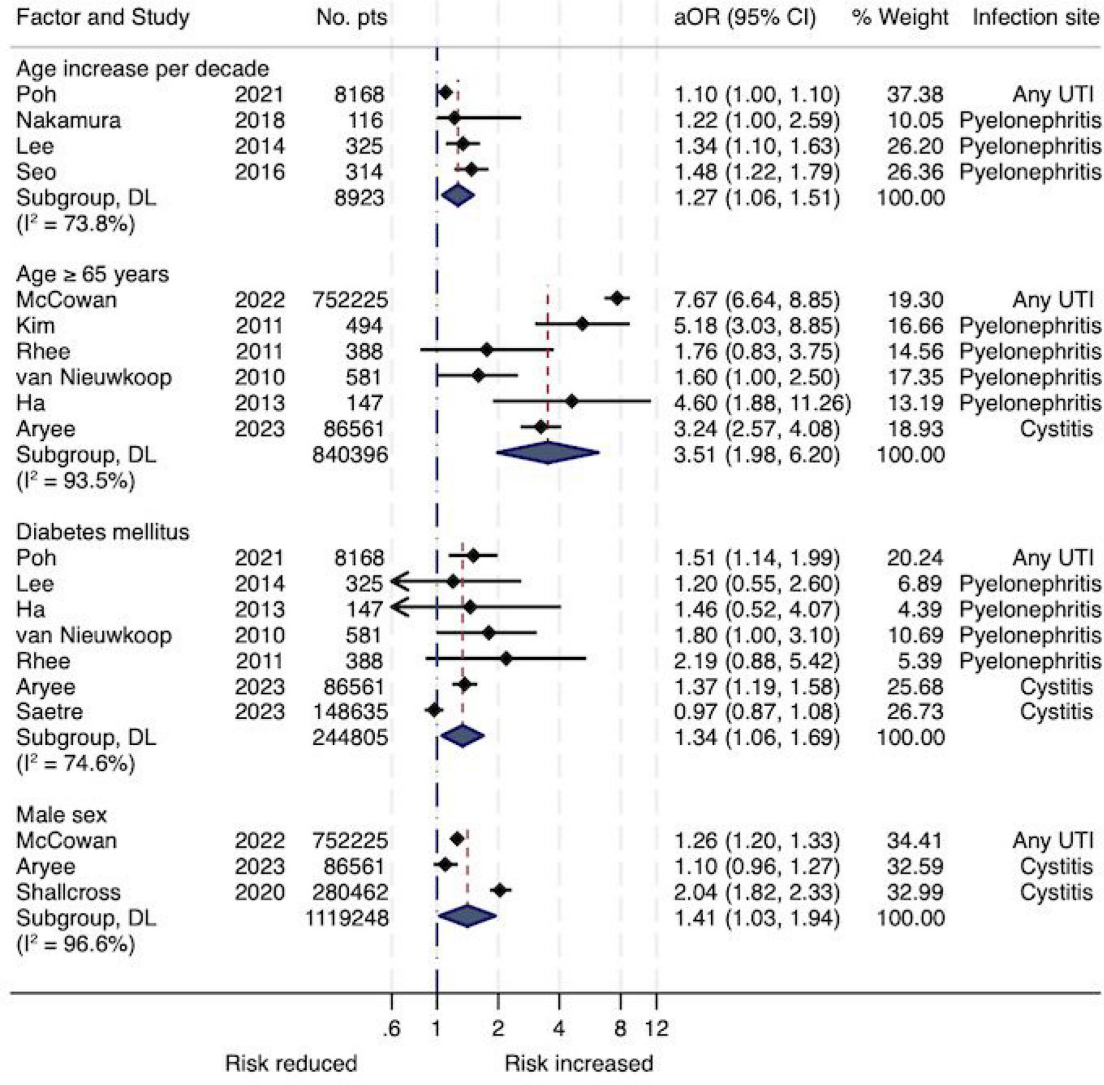
Forest plot showing the association between age, sex, diabetes mellitus, and hospitalisation from random-effects meta-analyses of adjusted odds ratios. aORs = adjusted odds ratios. DL = Der Simonian and Laird procedure. UTI = urinary tract infection.

**Figure 3.**
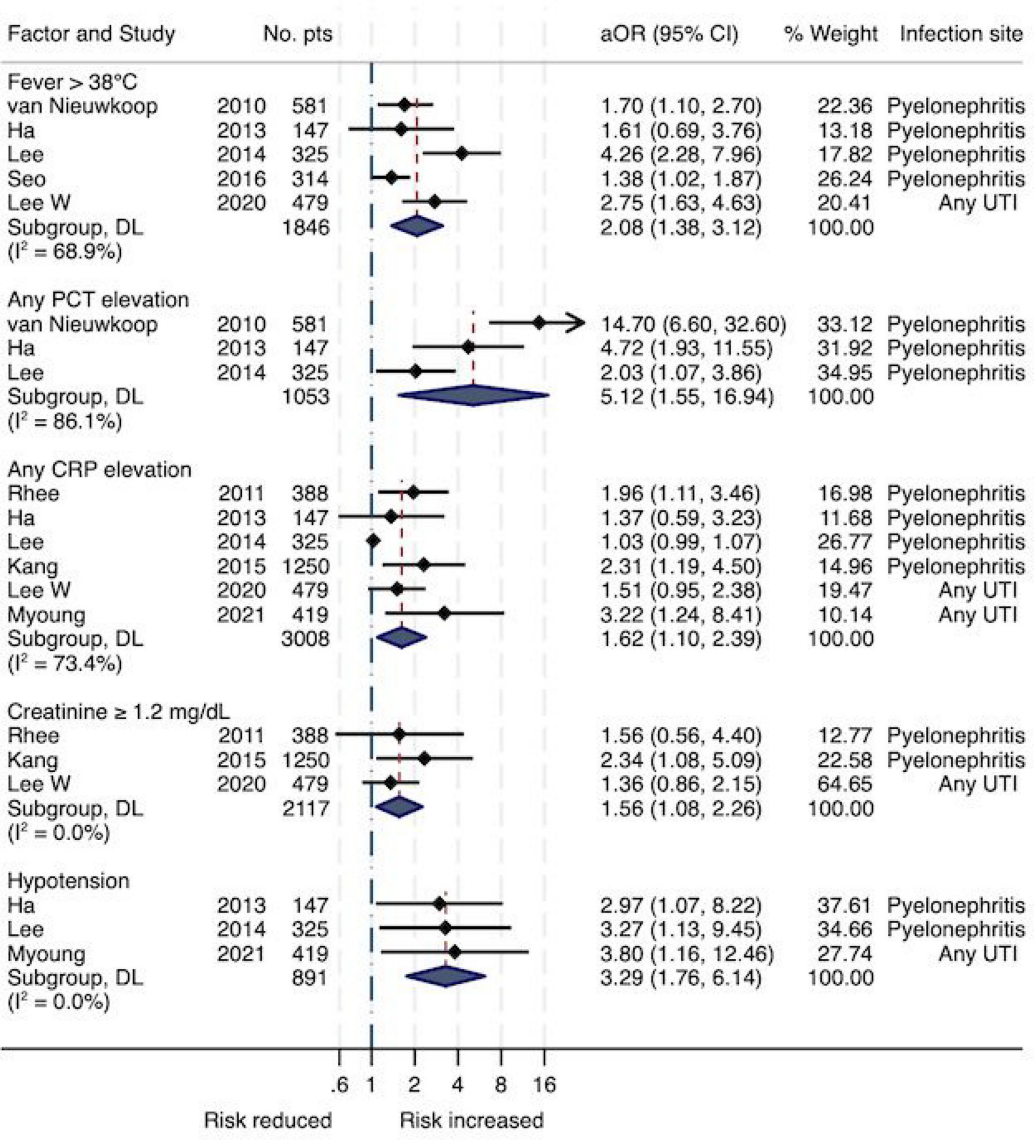
Forest plot showing the associations between signs and biomarkers and the outcome hospitalisation from random-effects meta-analyses of adjusted odds ratios. aORs = adjusted odds ratios; CRP = C-reactive protein; PCT = procalcitonin. UTI = urinary tract infection. DL = Der Simonian and Laird procedure.

Full results for meta-analysis of univariate RRs and aORs are shown in Appendix E. Forest plots for independent predictors are presented in Figures 2 and 3. Appendix B in the supplementary materials lists all variables from multivariable models. Forest plots of all performed analyses are provided in Appendix F.

#### Outcome of re-consultation

Thirteen studies reported on re-consultation as an outcome. Two included only patients with cystitis,^14,53^ five included patients with PN,^10,40,44,50,51^ and the remainder included patients with either condition.^13,57,58,61,63,64^ In the univariate random effects meta-analyses of RRs (Supplementary Table S8, Appendix E), factors associated with re-consultation were chronic kidney disease, benign prostate hyperplasia, recurrent UTI, catheter use, and any urinary tract abnormality. The meta-analysis of aORs (Supplementary Table S9, Appendix E) showed only increasing age to be associated with this outcome (aOR 1.18 per decade, 95% CI = 1.06 to 1.31).

#### Outcome of hospitalisation

Twenty-five studies reported risk factors for the outcome of hospitalisation. Four included patients with cystitis,^9,14,53,54^ 15 patients with PN,^12,15,16,38,39,41–49,52^ and the remainder included patients with either condition.^11,55,56,59–61,63^ In the univariate analyses (Supplementary Table S8, Appendix E), the factor most strongly associated with hospitalisation was a high PCT serum concentration, with the strongest association at a PCT >0.4–0.52 ng/ml (RR 10.6, 95% CI = 3.45 to 32.3).

The meta-analyses of aORs (Supplementary Table S9, Appendix E, Figures 2 and 3) confirmed the association, with an aOR of 5.12 (95% CI = 1.55 to 16.94) for any PCT elevation. Other independent predictors of hospitalisation in UTI patients included increasing age, either aged ≥65 years (aOR 3.51, 95% CI = 1.96 to 6.20) or per decade increase (aOR 1.27, 95% CI = 1.06 to 1.51); hypotension (aOR 3.29, 95% CI = 1.76 to 6.14); fever >38°C (aOR 2.08, 95% CI = 1.38 to 3.12); elevated CRP (aOR 1.62, 95% CI = 1.10 to 2.39); creatinine ≥1.2 mg/dl (aOR 1.56, 95% CI = 1.08 to 2.25); male sex (aOR 1.41, 95% CI = 1.03 to 1.94); and diabetes mellitus (aOR 1.34, 95% CI = 1.06 to 1.69). As shown in Figures 2 and 3, among those risk factors, age ≥65 years, diabetes, male sex, and CRP elevation are associated with hospitalisation in both, patients with cystitis and PN. Hypotension, fever, and elevated PCT or creatinine are associated with hospitalisation in patients with PN only.

#### Outcome of mortality

Four studies reported on mortality.^11,55,62^ One included only patients with cystitis , and the remainder included patients with either cystitis or PN. Only male sex was assessed by more than two studies,^11,55,62^ and meta-analysis of RRs showed a non-significant association with mortality (RR 1.36, 95% CI = 0.95 to 1.94, Supplementary Table S8, Appendix E).

No studies were available for meta-analyses of aORs, as only one large study on patients aged ≥65 years with acute cystitis provided adjusted estimates.^54^ In these patients, all-cause mortality was independently associated with male sex (aOR 1.75, 95% CI = 1.67 to 1.89); age per 5 years increase (aOR 1.62, 95% CI = 1.59 to 1.65); a higher Charlson Comorbidity Index (aOR 1.52, 95% CI = 1.46 to 1.59); hospitalisation and emergency attendance in the previous month (aOR 1.43, 95% CI = 1.29 to 1.58 and aOR 1.30, 95% CI = 1.16 to 1.47, respectively); smoking (aOR 1.35, 95% CI = 1.20 to 1.51); antibiotics in the previous month (aOR 1.25, 95% CI = 1.17 to 1.33); and no antibiotics at initial consultation (aOR 1.17, 95% CI = 1.09 to 1.26, Supplementary Table S9, Appendix E).^54^ No study reported adjusted results for patients with PN.

## Discussion

### Summary

Older age was the only independent predictor of re-consultation in patients with UTI. Elevated PCT emerged as the strongest risk factor for hospitalisation, with additional predictors including advanced age (aged ≥65 years and per decade increase), hypotension, fever (>38°C), elevated CRP, creatinine (≥1.2 mg/dl), male sex, and diabetes. Analyses by infection site suggest that high PCT and creatinine, hypotension, and fever are particularly associated with hospitalisation in patients with PN rather than cystitis. Table 1 summarises these findings. For mortality, a single study identified key predictors in adults aged ≥65 years with cystitis, male sex, older age, comorbidities, hospitalisation or emergency attendance or antibiotics in the previous month, and no antibiotics at initial consultation.

**Table 1.** Summary of main results: independent predictors for re-consultation and hospitalisation (primary outcomes) in patients with UTI (cystitis and pyelonephritis), and pyelonephritis only. Within each category, risk factors are listed in order of strength of association

Re-consultation variable (aOR)	**Hospitalisation** variable (aOR)
**Cystitis and pyelonephritis**	
**Age, per decade increase** (1.18)	**Age ≥65 years** (3.51) **Any CRP elevation** (1.62) **Male sex** (1.41) **Diabetes mellitus** (1.34) **Age, per decade increase** (1.27)
**Pyelonephritis only**	
N.A.	**Any PCT elevation** (5.12) **Low systolic BP** (3.29) **Fever >38**°**C** (2.08) **Creatinine ≥1.2 mg/dl** (1.56)

aOR = adjusted odds ratio. BP = blood pressure. CRP = C-reactive protein. N.A. = not available for meta-analysis (< three studies). PCT = procalcitonin

### Strengths and limitations

The primary strengths of this study are the comprehensive search strategy, meta-analysis of both univariate and multivariate associations, and the breadth of patient-relevant outcomes and risk factors. In addition, our focus on outpatient settings makes the results relevant for primary care and ED physicians. There were several limitations. First, we were not able to identify a common set of adjustment factors across the reported multivariable models, so residual confounding is likely. Second, most studies were retrospective, and some were registry based. While this approach enabled the study of risk factors for relatively rare outcomes, it increased the likelihood of misclassification of the exposure and outcome. We mitigated this by not including retrospective studies of symptoms as risk factors. Third, some of the studies included both patients with cystitis and with PN. This made it difficult in some instances to attribute a certain risk factor to a definite patient population. We mitigated this performing meta-analyses overall and separately for PN studies and by reporting findings of primary studies separately. Fourth, we cannot exclude that publication bias may have distorted the observed associations. However, adhering to current recommendations, we avoided exploring publication bias formally as our meta-analyses were too small, and formal investigation could, in these cases, lead to even more bias.^65^ Fifth, between-study heterogeneity was high for some associations and the limited number of studies did not allow us to explore causes of heterogeneity.

### Comparison with existing literature

In keeping with the National Institute for Health and Care Excellence (NICE) and European Association of Urology (EAU) guidelines^4,18^ and previous studies,^66^ we found that increasing age, male sex, and diabetes mellitus are associated with a more complicated course in both patients with acute cystitis and those with PN. However, in adjusted analyses, other factors classically deemed to be risk factors for adverse UTI outcomes, such as immunodeficiency, catheter use, urinary tract abnormalities, and other comorbidities were not associated with poor prognosis. These findings may reflect the lack of power of our meta-analyses and the exclusion of some patient groups in individual studies. Another reason could be that three^9,53,54^ out of four of the included studies on cystitis included some individuals with asymptomatic bacteriuria, as the cohorts of included patients were selected using Read codes of routine data. This, in turn, could have confounded the associations reported in individual studies, as well as our results. However, our findings seem to suggest that current recommendations and UTI risk stratification systems are not based on reproducible empirical evidence, rather on expert consensus and pathophysiological reasoning, as recently noted elsewhere.^8,17^ This is problematic, especially for a very frequent condition such as acute cystitis in women, for which many clinical guidelines allow for alternatives to antibiotics if the patient is at ’low risk’ of complications.^17,18,24^ While this recommendation seems reasonable, as the absolute risk of complications is low,^67^ our study shows that the knowledge on which factors may identify this ’low risk’ category for a complicated course in patients with cystitis is still very limited.

Elevated serum CRP and, in patients with PN, PCT are associated with an increased risk of hospitalisation. Two studies, including patients with acute cystitis, found that an elevated serum CRP was associated with a more severe disease course, but the evidence is limited for this patient population.^60,61^ CRP-guided management has proven to be effective in improving the management of respiratory tract infections.^68–71^ Such point-of-care test guided approaches might also be beneficial in patients with UTI. To date, only one small randomised controlled trial (RCT) has evaluated a PCT and pyuria-based algorithm on antibiotic use in UTI, showing promising results.^72^

### Implications for research and practice

This study represents the most comprehensive evidence on outpatient relevant risk factors for adverse outcomes in adults’ UTI. However, studies focusing on acute cystitis are limited, and longitudinal studies linked to secondary care and microbiological data for outcome ascertainment are needed. Such studies could aid in developing and validating clinical risk scores, currently lacking for both cystitis and PN. Researchers should incorporate identified factors into prediction models, and prospective studies are needed to compare management strategies using risk scores and biomarkers like CRP and PCT, given the growing availability of point-of-care tests in ambulatory settings.

Notably, no outpatient PN study provided adjusted mortality estimates, revealing a research gap requiring further studies.

Until stronger evidence is available, guidelines should prioritise risk factors with the strongest associations with adverse outcomes (older age, hypotension, fever, elevated inflammatory biomarkers, creatinine, male sex, diabetes) to define complicated cases. Clinicians may consider using these factors to identify patients needing closer follow-up.

## Trial registration number

PROSPERO: CRD42022355676.

## Data

Raw data and code for analysis are available upon reasonable request from the corresponding author.
